# Targeted multiplex validation of CSF proteomic biomarkers: implications for differentiation of PCNSL from tumor-free controls and other brain tumors

**DOI:** 10.3389/fimmu.2024.1343109

**Published:** 2024-08-01

**Authors:** Jingjing Ma, Zhiguang Lin, Yaqi Zhang, Yun Ding, Qiming Tang, Yufeng Qian, Bo Jin, Ruben Y. Luo, Wei-Li Liao, Sheeno Thyparambil, Zhi Han, C. James Chou, James Schilling, Qing Li, Mengxue Zhang, Yunan Lin, Yan Ma, Karl G. Sylvester, Seema Nagpal, Doff B. McElhinney, Xuefeng B. Ling, Bobin Chen

**Affiliations:** ^1^ Department of Hematology, Huashan Hospital, Fudan University, Shanghai, China; ^2^ College of Automation, Guangdong Polytechnic Normal University, Guangzhou, China; ^3^ Research and Development, mProbe Inc. Palo Alto, CA, United States; ^4^ Department of Pathology, Stanford University School of Medicine, Stanford, CA, United States; ^5^ Department of Biomedical Data Science, Stanford University School of Medicine, Stanford, CA, United States; ^6^ Department of Surgery, Stanford University School of Medicine, Stanford, CA, United States; ^7^ Department of Neurology and Neurological Sciences, Stanford University School of Medicine, Stanford, CA, United States; ^8^ Departments of Cardiothoracic Surgery and Pediatrics (Cardiology), Stanford University School of Medicine, Stanford, CA, United States

**Keywords:** primary central nervous system lymphoma (PCNSL), diffuse large B-cell lymphoma (DLBCL), cerebrospinal fluid (CSF), targeted proteomics, liquid chromatography tandem-mass spectrometry (LC-MS/MS)

## Abstract

**Introduction:**

Primary central nervous system lymphoma (PCNSL) is a rare type of non-Hodgkin’s lymphoma that affects brain parenchyma, eyes, cerebrospinal fluid, and spinal cord. Diagnosing PCNSL can be challenging because imaging studies often show similar patterns as other brain tumors, and stereotactic brain lesion biopsy conformation is invasive and not always possible. This study aimed to validate a previous proteomic profiling (PMID: 32610669) of cerebrospinal fluid (CSF) and develop a CSF-based proteomic panel for accurate PCNSL diagnosis and differentiation.

**Methods:**

CSF samples were collected from patients of 30 PCNSL, 30 other brain tumors, and 31 tumor-free/benign controls. Liquid chromatography tandem-mass spectrometry targeted proteomics analysis was used to establish CSF-based proteomic panels.

**Results:**

Final proteomic panels were selected and optimized to diagnose PCNSL from tumor-free controls or other brain tumor lesions with an area under the curve (AUC) of 0.873 (95%CI: 0.723-0.948) and 0.937 (95%CI: 0.807- 0.985), respectively. Pathways analysis showed diagnosis panel features were significantly enriched in pathways related to extracellular matrices-receptor interaction, focal adhesion, and PI3K-Akt signaling, while prion disease, mineral absorption and HIF-1 signaling were significantly enriched with differentiation panel features.

**Discussion:**

This study suggests an accurate clinical test panel for PCNSL diagnosis and differentiation with CSF-based proteomic signatures, which may help overcome the challenges of current diagnostic methods and improve patient outcomes.

## 1 Introduction

Primary central nervous system lymphoma (PCNSL) is a type of non-Hodgkin’s B-cell (NHL) lymphoma that belongs to the diffuse large B-cell lymphoma type (DLBCL) in >90% of cases. Unlike other types of lymphomas, it is confined to the central nervous system (CNS), including the brain parenchyma, eyes, cerebrospinal fluid (CSF), or spinal cord with the absence of systemic involvement at the time of diagnosis, and this makes it an “immune-privileged” form of lymphoma. PCNSL is considered a rare type of lymphoma and can be challenging to diagnose and treat due to its unique location and presentation (1). The incidence of PCNSL is relatively low, occurring at a rate of approximately 0.47 per 100,000 person-years. PCNSL accounts for approximately 4% to 6% of lymphomas that arise outside of the lymph nodes, and 4% of newly diagnosed CNS tumors (2, 3). Compared to systemic DLBCL, PCNSL exhibits a more invasive growth pattern, can be associated with infiltration of the vitreous and chorioretina, and have a less favorable prognosis (4, 5).

Despite the development of advanced radiological imaging techniques, such as whole-body positron emission tomography/computed tomography (PET-CT) and magnetic resonance imaging (MRI), stereotactic brain lesion biopsy remains the gold standard for the diagnosis of PCNSL and differentiation from other brain tumors. However, brain biopsy is an invasive procedure that carries risks of complications, such as functional impairment and intracranial hemorrhage, particularly when attempting to reach deep tumor sites. Furthermore, brain biopsy may not always provide a definitive diagnosis due to sampling errors, insufficient tumor tissue, or pre-operative steroid use (6–8). Given the challenges associated with brain biopsy and the urgent clinical need for accurate and timely diagnosis of PCNSL, there is a growing interest in exploring less invasive and efficient diagnostic and differentiation tests. Such tests would not only help to avoid the risks and limitations of brain biopsy but also enable early detection and timely treatment, which can have a significant impact on patient outcomes. Therefore, developing non-invasive biomarker-based diagnostic approaches, such as those based on proteomic or transcriptomic analysis of cerebrospinal fluid, represents a promising avenue for improving the diagnosis and differentiation of PCNSL.

Liquid biopsy, which involves the analysis of biofluids to detect biomarkers of cancer, is becoming an increasingly important tool in neuro-oncology. While blood is often used as the primary liquid biopsy source for other tumor types, CSF has been suggested to be an ideal liquid biopsy source for brain tumors. This is due to the fact that CSF has closer proximity to the tumor microenvironment in the CNS and is not affected by the blood-brain barrier. Compared to serum, background levels of proteins and DNA or RNA are limited, indicating reduced interference in the detection of genuine biomarkers. As a result, CSF can receive leakage molecules from neural tissues through passive apoptotic/necrotic processes or active secretion such as ectodomain shedding or vesicular transportation (9–11). Recent studies have shown promising results in identifying potential diagnostic and prognostic biomarkers for PCNSL in the CSF. Cytokines such as interleukin IL-6, IL-10, CXCL-12, and CXCL-13 have been found to be increased in the CSF of PCNSL patients, suggesting their potential as biomarker candidates (12–15). In addition to cytokines, expression levels of B-cell marker, CD19 (16), CD27 (17) and some immune responses maker such as neopterin (18) and osteopontin (19) have also been reported to be up-regulated and suggested as potential diagnostic markers for PCNSL.

Although numerous CSF studies have been performed to identify and develop a minimally invasive diagnostic test for PCNSL, inadequate cohort sizes and limited focus on certain molecular processes may overlook additional potential diagnostic biomarkers. Additionally, other brain tumor lesions, such as glioblastoma or demyelination, that display similar symptoms and imaging results are often excluded as comparison groups, which could hinder the identification of unique biomarkers for PCNSL. These challenges emphasize the need for more extensive and inclusive studies to develop a reliable and efficient diagnostic test for PCNSL. Recently, a recent report of proteomic profiling study of PCNSL, secondary central nervous system lymphomas (SCNSL), multiple sclerosis (MS), glioma, other tumors and tumor-free controls discovered a large set of proteomic signatures using a cohort of European patients (PMID: 32610669) (20). To validate the effectiveness and generalizability of the signature molecules from this study, we conducted an extensive liquid chromatography-mass spectrometry (LC-MS/MS) targeted proteomic analysis in a cohort of Chinese population. The biomarkers identified through LC-MS/MS were used to construct proteomic panels that could differentiate PCNSL from both tumor-free controls and other brain tumor entities in CSF. We also investigated the underlying pathophysiological processes that cause the observed differences in protein biomarkers in PCNSL patients compared to other brain tumors and tumor-free controls.

## 2 Materials and methods

### 2.1 Patients and CSF collection

We retrospectively identified patients who had space-occupying brain lesions treated by the neurosurgery, neurology, infection, and hematology services in our institution from December 2021 to December 2022. These patients required routine CSF analysis, MRI contrast-enhanced scans, PET-CT scans, and/or stereotactic brain lesion biopsy for pathologic findings ultimately confirmed as PCNSL (n=30), other brain tumors (n=30), or non-malignant disease controls (n=31). All patients provided written informed consent for the participation in this study. All experiments were conducted in accordance with the Declaration of Helsinki and Good Clinical Practice guidelines and were approved by the Ethics Committee of our institution.

The CSF collection procedure followed standard operating procedures, where CSF was collected by lumbar puncture at room temperature, and the first 10 drops were discarded to avoid blood contamination. The collected CSF was immediately centrifuged at 3000 rpm at 4°C for 10 minutes to remove cell debris, and 1 mL aliquots of the supernatant were stored at -80°C for further assays. The entire procedure was completed within 30 minutes. For analysis of PCNSL diagnosis, 61 patients were included, consisting of 31 non-malignant disease controls and 30 PCNSL patients. For analysis of differentiation between tumor types, 60 patients were included, consisting of 30 non-PCNSL brain tumor controls and 30 PCNSL patients.

### 2.2 CSF sample pre-processing for proteome analysis

To analyze the cerebrospinal fluid CSF samples, 10 μL was collected from each patient and thawed on ice from -80°C. To prepare the samples for SDS-PAGE separation, they were diluted 4-fold with running buffer and boiled for 5 minutes. Then, 20 μg of each sample was loaded onto a 4%-20% gradient gel from BioRad and run for 65 minutes at 120V. Once SDS-PAGE separation was complete, the gel was stained and divided into 10 evenly-sized bands based on the molecular size marker. Each band was then transferred to a 1.5 mL Eppendorf tube, de-stained, and dried. To proceed with analysis, the gel bands were denatured, alkylated, and digested with trypsin for 16 hours at 37°C. After digestion, the peptides were extracted from the gel with 100 µL of 60% ACN/0.1% FA solution, and this process was repeated 3 times. The extracted peptides were combined, freeze-dried, and then dissolved in 20 µL of 0.1% FA buffer for LC-MS analysis. Finally, the concentration of peptides in the reconstituted sample was determined using a microBCA assay.

### 2.3 Liquid chromatography coupled mass spectrometric proteomic analysis

LC-MS/MS analysis was performed using a Q ExactiveTM Plus Hybrid Quadrupole-OrbitrapTM mass spectrometer from Thermo. Five microliters of sample were injected, and peptides were separated and detected using an EASY-SprayTM-C18 column (50cm x 75 μm, 2µm, Thermo, ES803A) coupled with electrospray ionization. The separation was carried out using a 60-minute reversed-phase gradient containing 0.1% formic acid in water as mobile phase A and 0.1% formic acid in acetonitrile as mobile phase B. Full MS was scanned from 300-1650 m/z at 60000 resolution, while MS2/SIM was acquired with inclusion lists of significant proteins from the reference at 15000 resolution and a 1.5 m/z isolation window. The resulting MS data were analyzed using Proteome DiscoveryTM (ver 2.1, Thermo), and the results were exported for further statistical analysis.

### 2.4 Bioinformatics and statistical analysis

For diagnosis analysis, we obtained two gene expression datasets, GSE11392 (Pathway analysis of primary central nervous system lymphoma (PCNSL), PMID: 18684868) and GSE25297 (Genome-wide gene expression comparison (primary central nervous system lymphoma (PCNSL) vs normal lymph node, PMID: 21088137), from Gene Expression Omnibus (GEO) for the diagnosis analysis. These combined datasets included a total of 20 PCNSL and 7 normal lymph node tissues. For the differentiation analysis, we obtained 6 gene expression datasets from GEO: GSE34771 (Expression data from primary central nervous system lymphoma (PCNSL) patients, PMID: 22908096),GSE61578 (Gene Expression and HD-SNP6.0 data from Primary Testicular (PTL), Primary Central Nervous System Lymphoma (PCNSL) and Primary Mediastinal B-cell Lymphoma (PMLBCL), PMID: 26702065), GSE43378 (Expression data from glioma patients, PMID: 23745793),GSE124145 (Basic characteristics of glioma stem cell and human glioblastoma, PMID: 30711935), GSE60184 (UCSD GBM Data Set, PMID: 25277177) and GSE36245 (Gene expression data from glioblastoma tumor samples, PMID: 23079654). These combined datasets included a total of 49 PCNSL and 122 glioblastoma tissues.

### 2.5 Differential protein analysis

10,435 peptides reported from **Supplementary Table S2** of Waldera-Lupa et al., 2020 was re-analyzed. p<0.05 and AUC≥0.7 were used as criteria to screen the univariant analysis results from PCNSL vs. Control in the table. Significantly differentiated peptides were identified and corresponding unique proteins were selected as biomarker candidates. The same criteria were used to screen the univariant analysis results from PCNSL vs. Glioma and PCNSL vs. Tumor in the table.

We considered statistical significance to be P < 0.05 unless stated otherwise. Groups were compared using 2-tailed t-test and Mann-Whitney U test. Logistic regression was used to create receiver operating characteristic curves (ROC) and area under the ROC curve (AUC) was measured for each detected target protein.

### 2.6 Stratification model

We evaluated 8 commonly used clinical algorithms to optimize data analysis and class separation: random forest (RF), logistic regression, multilayer perceptron (MLP), support vector machine (SVM), XGB, k-nearest neighbors (KNN), lasso regression, and elastic network (EN). We compared 3 different cross-validation methods: “leave one out”, 5-fold, and 10-fold and chose “leave one out” for the final cross-validation and statical analysis. The final validation phase determined the optimal classification based on the best performance according to ROC AUC.

## 3 Results

### 3.1 Study workflow for biomarker panel validation for PCNSL diagnosis and differentiation

**Figure 1:**
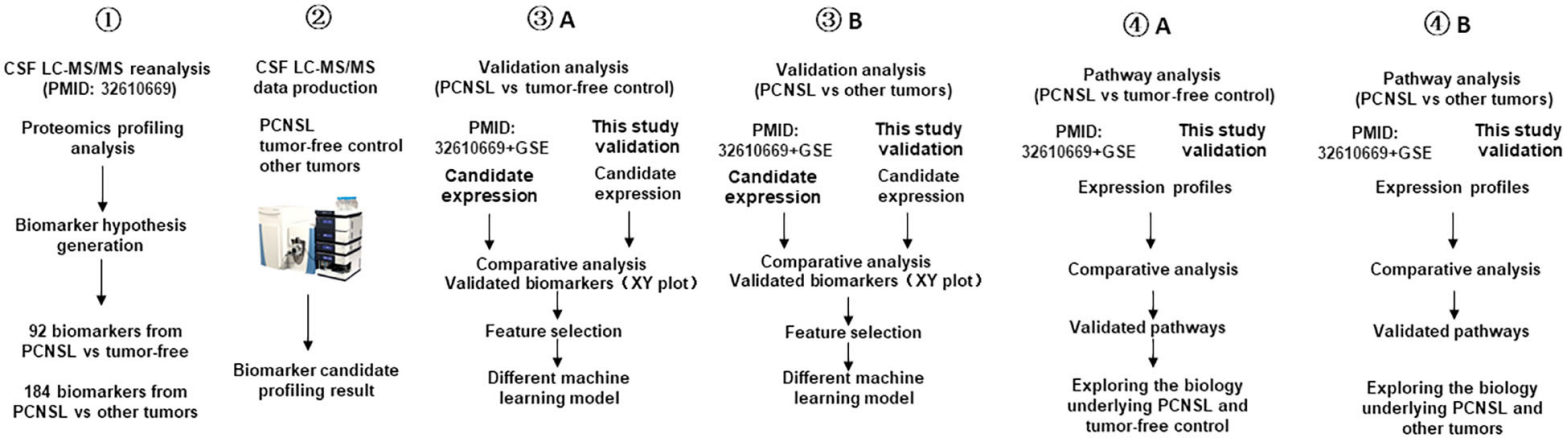
Study workflow diagram. This workflow outlines the design of the PCNSL diagnosis and differentiation panel development. It involves the re-analysis of proteomics profile data (PMID: 32610669), targeted proteomics production in the CSF, validation and development of diagnosis and differentiation biomarkers, and pathway analysis to investigate the underlying biology of PCNSL.

**Figure 1** illustrates the overall design of our study, which involves re-analysis of CSF proteomics data from a reference study, targeted proteomics analysis of our own CSF cohort, validation of potential biomarkers for PCNSL diagnosis and differentiation from other brain tumors, and pathway analysis for better understanding of the underlying biology.

To begin with, we validated potential biomarkers by re-analyzing the CSF proteomics profiling results obtained by Stühler’s group (PMID: 32610669), who used quantitative MS to profile the proteome signatures of CSF from patients with different types of brain tumors as well as those without tumors. We then performed targeted proteomics using LC-MS/MS on our own cohort of PCNSL (n=30), other brain tumors (n=30), and tumor-free controls (n=31). We validated the differential expression of the biomarker candidates based on the ROC AUC and categorized the validated biomarkers based on the ROC AUC in the tissue transcription profile. After validating the biomarkers, we developed PCNSL diagnosis and differentiation panels and compared various modeling algorithms to optimize the stratification performance. Finally, we investigated the differential pathways containing the protein biomarkers from the two stratification panels and explored the potential molecular mechanisms underlying PCNSL pathology.

### 3.2 PCNSL validation cohort patients’ characteristics

To identify stratification biomarkers of PCNSL from tumor-free controls and other brain tumor patients, we collected CSF from three groups of patients: those diagnosed with PCNSL, other brain tumors including glioma, brain metastases (lung cancer, breast cancer, etc.), as well as tumor-free individuals. The demographic and clinical characteristics of the validation cohort, including 30 PCNSL, 30 other brain tumors, and 31 tumor-free controls, are presented in **Table 1**. We found no significant differences in demographic or basic CSF clinical characteristics between PCNSL and tumor-free controls, except that the age of the tumor-free controls was younger. Furthermore, there were no differences in demographic and clinical characteristics between PCNSL and other brain tumor samples. However, we did observe significantly higher levels of IL-10 and IL-6 in CSF samples from PCNSL patients.

**Table 1:** Demographics of the CSF validation cohort.

Characteristic	PCNSL (n=30)	Tumor-free control (n=31)	Other brain tumors (n=30)	*P*-value (χ2, PCNSL vs Tumor-free control)	*P*-value (χ2, PCNSL vs other brain tumors)
Age					
Median(range)	57 (26-80)	48 (19-72)	58 (34-69)	0.006825	0.681
Sex					
Male	18	12	11	0.096	0.0705
Female	12	19	19		
CSF nucleated cells					
(0-8)*10^9/L	20	26	20	0.119	1
>8*10^9/L	10	5	10		
CSF protein					
>0.45g/L	18	11	20	0.055	0.592
≤0.45g/L	12	20	10		
CSF tumor cells					
Positive	19	0	18	<0.001	0.791
Negative	11	31	12		
CSF IL-10					
Elevated	22	n.t	4	NA	<0.001
Normal	8	n.t	26		
CSF IL-6					
Elevated	20	n.t	12	NA	0.0384
Normal	10	n.t	18		
Brain parenchyma lesion					
Yes	28	11	27	<0.001	0.640
No	2	19	3		

### 3.3 Discovery of differential expressed proteins biomarkers in PCNSL, tumor-free controls and other brain tumors

In the recent study by Stühler’s group, proteomic profiles were analyzed in the CSF collection of 6 clinical groups including PCNSL, SCNSL, MS, glioma, other tumors, and tumor-free controls. We utilized their differential proteome analysis results and re-analyzed them to compare PCNSL with tumor-free controls. Using p<0.05 and ROC AUC ≥0.7 as criteria, from PCNSL vs. Control data set, we found 198 peptides corresponding to 92 unique protein biomarker candidates. We used the same criteria to screen the univariant analysis results from PCNSL vs. Glioma and PCNSL vs. Tumor dataset, we found 270 and 94 peptides, respectively. Then we took the union of these two sets to get 364 peptides, which corresponded to 184 unique protein biomarker candidates for stratifying PCNSL from other brain tumors including glioma.

### 3.4 Validation of proteins biomarkers between PCNSL and tumor-free controls

**Figure 2:**
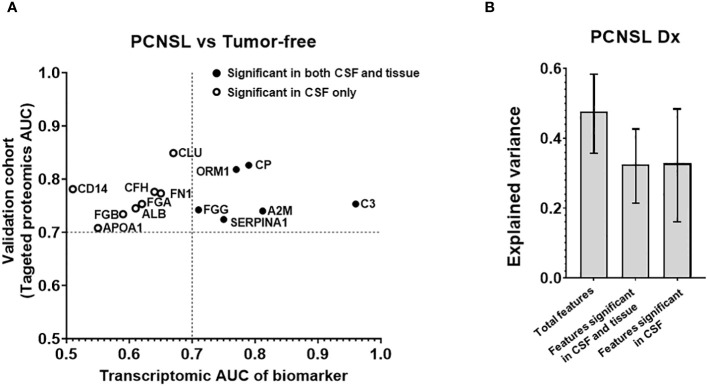
Analysis of the targeted proteins from reference biomarker panel of PCNSL diagnosis in validation cohort. **(A)** ROC AUC values from the transcriptomic data and our targeted proteomics for confirmed protein biomarkers. Proteins that were only significant in CSF in our validation cohort are represented by a hollow circle. Solid-filled circles represent proteins that were significant in both CSF and tissue corresponding gene expression datasets. **(B)** Explained variance scores of the 14 proteins from the complete diagnosis panel, a 6-protein subset showing significance in both CSF and tissue, and an 8-protein subset showing significance in CSF only.

To validate the biomarkers that differentiate PCNSL from tumor-free controls, we conducted a targeted proteomics analysis. **Figure 2A** illustrates that out of the initial 92 biomarker candidates, 14 proteins were confirmed to exhibit significant separation of PCNSL from tumor-free controls based on ROC AUC. Additionally, **Supplementary Figure 1** showed 14 candidate biomarker peptides’ concentration in PCNSL and tumor-free controls, using mass spectrometry intensity to represent the concentration of candidate biomarker peptides.

To investigate whether the observed differences in CSF levels of these 14 proteins were attributable to pathological changes in brain lesions, we assembled a tissue gene expression cohort and compared the expression of these 14 biomarkers between PCNSL lymphoma and normal lymph node tissues. **Table 2A** shows that the tissue cohort for PCNSL diagnosis combined two GSE tissue gene expression datasets, comprising 20 PCNSL and 7 normal tissues. We calculated the ROC AUC for the corresponding gene of each of the 14 biomarkers between PCNSL and normal tissues. **Supplementary Figure 2** showed boxplots of the 14 candidate biomarker concentration in PCNSL and tumor-free controls, represented by normalized gene expression value from microarray. Six genes were found to be significant in both the CSF and tissue cohorts (ROC AUC>0.7 in both transcriptomic data and targeted proteomics data, depicted in black in **Figure 2A**): Ceruloplasmin (CP), Orosomucoid 1 (ORM1), Alpha-2 Macroglobulin (A2M), Fibrinogen Gamma Chain (FGG), Complement C3 (C3) and Serpin Family A Member 1 (SERPINA10). The other eight genes were found to be significant in CSF only (ROC AUC>0.7 in targeted proteomics data but not in transcriptomic data, depicted in circle in **Figure 2A**): CD14 Molecule (CD14), Fibrinogen Beta Chain (FGB), Clusterin (CLU), Apolipoprotein A1 (APOA1), Fibronectin 1 (FN1), Complement Factor H (CFH), Albumin (ALB) and Fibrinogen Alpha Chain (FGA).

**Table 2:** Demographic table of GSE datasets.

A PCNSL Dx							
Dataset	Study	PCNSL (n)	non-CNS DLBCL (n)	Normal	Number of Transcripts	Microarray Platform	PMID
**1**	GSE11392	13	30	/	41059	GPL6848	18484868
**2**	GSE25297	7	/	7	36922	GPL6480	21088137
B PCNSL Diff							
**Dataset**	**Study**	**PCNSL (n)**	**GBM**	**Normal**	**Number of Transcripts**	**Microarray Platform**	**PMID**
**1**	GSE34771	34	/	/	54675	GPL570	22908096
**2**	GSE61578	15	/	/	54675	GPL570	26702065
**3**	GSE43378	/	50	/	54675	GPL570	23745793
**4**	GSE124145	/	3	/	54675	GPL570	30711935
**5**	GSE60184	/	23	/	54675	GPL570	25277177
**6**	GSE36245	/	46	/	54675	GPL570	23079654

To further assess the contribution of potential tissue leakage biomarkers (n=6, significant in tissues) and host response biomarkers (n=8, not significant in tissues), we compared the explained variance scores shown in **Figure 2B**. The total explained variance score for all 14 features was 0.477, while the scores for features from tissue and host response were 0.325 and 0.328, respectively. These results suggest that both tissue and host response features contribute equally to the differentiation of PCNSL from tumor-free controls.

**Figure 3:**
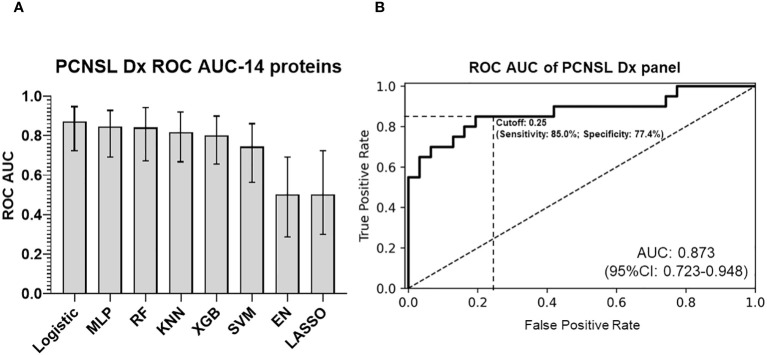
PCNSL diagnosis panel with 14 validated proteomic biomarkers. **(A)** Comparison of different modeling algorithms for PCNSL diagnosis stratification performance based on ROC AUC. **(B)** ROC AUC curve of the final 14-protein panel for PCNSL diagnosis using the logistic regression algorithm. The modeling algorithms compared include MLP (Multilayer Perceptron), RF (Random Forest), KNN (K-Nearest Neighbors), XGB (XGBoost), SVM (Support Vector Machine), and EN (Elastic Net linear regression). Dashed lines showed the optimized sensitivity and specificity at the chosen threshold 0.25.

After validating the 14 differentially expressed protein biomarkers, we compared various algorithms to develop a diagnosis model for CSF (with “leave-one-out” cross validation). Logistic regression of the 14-biomarker panel showed the best ROC AUC for separating PCNSL from tumor-free controls, AUC 0.873 (95%CI: 0.723-0.948), as shown in **Figures 3A, B**. We further evaluated the stratification performance of potential tissue leakage biomarkers and host response biomarkers. ROC AUC of these two groups were 0.660 (95%CI: 0.464-0.815) and 0.863 (95%CI: 0.704-0.941) respectively, as shown in **Supplementary Figure 3**.

The importance of each protein from diagnosis panel is depicted in **Supplementary Figure 4**. Our panel of 14 biomarkers provided a better diagnostic accuracy for PCNSL (PCNSL diagnosis from other brain lesions) compared to CT or MRI, with ROC AUC values of 0.726 for CT, 0.785 for MRI, and 0.845 for CT and MRI (21).

### 3.5 Validation of proteins biomarkers between PCNSL and other brain tumors

**Figure 4:**
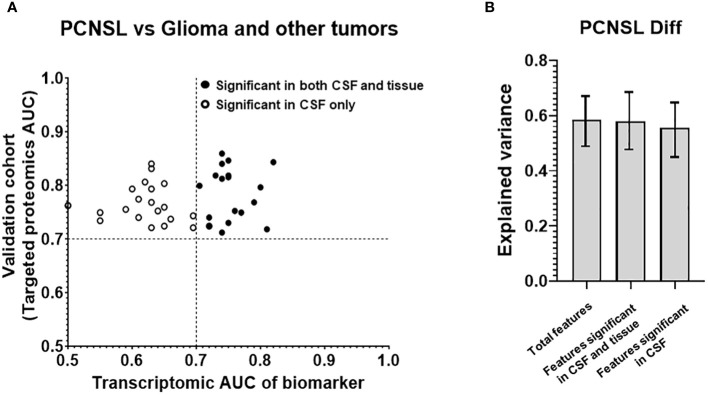
Analysis of the targeted proteins from reference biomarker panel of PCNSL differentiation in validation cohort. **(A)** ROC AUC values from the transcriptomic data and our targeted proteomics for confirmed protein biomarkers. Proteins that were only significant in CSF in our validation cohort are represented by a hollow circle, while those that were significant in both CSF and tissue are represented by a black-filled circle. **(B)** Explained variance scores for 39 proteins in the complete diagnosis panel, as well as subsets of 18 proteins showing significance in both CSF and tissue and 21 proteins showing significance in CSF only.

In addition to PCNSL diagnosis, differentiating PCNSL from other brain tumor lesions is also a key unmet clinical need due to the challenge of distinguishing between different brain tumors with similar CT/MRI imaging patterns. We used the proteomic profiling results from Stühler’s group on glioma and other tumors and compared them with PCNSL samples. We discovered 184 protein features that were differentially expressed with statistical significance (p<0.05, ROC AUC>0.7) between PCNSL and other brain tumors. Using our targeted proteomics analysis on the collected CSF of 30 patients with PCNSL and 30 with other brain tumors, 39 potential protein biomarkers were confirmed for significant PCNSL differentiation, with ROC AUC >0.7 in our CSF cohort, as shown in **Figure 4A.** Additionally, **Supplementary Figure 5** showed these 39 candidate biomarker peptides’ concentration in PCNSL and tumor-free controls, using mass spectrometry intensity to represent the concentration of candidate biomarker peptides.

To investigate which features may have originated from tissue leakage, we created a tissue cohort, as shown in **Table 2B**. This cohort consisted of 6 GSE tissue gene expression datasets, which included a total of 49 PCNSL lymphoma tissues and 122 glioblastoma tissues. The corresponding gene for the 39 biomarkers was used to calculate the ROC AUC between PCNSL and other brain tumors. **Supplementary Figure 6** showed boxplots of the 39 candidate biomarker concentration in PCNSL and other brain tumors, represented by normalized gene expression value from microarray. 18 genes were found to be significant in the tissues (ROC AUC>0.7 in both transcriptomic data and targeted proteomics data, as shown in black in **Figure 4A** including: Complement Factor H (CFH), Clusterin (CLU), CD14 Molecule (CD14), Serpin Family A Member 1 (SERPINA1), Retinol Binding Protein 4 (RBP4), Prostaglandin D2 Synthase (PTGDS), Lumican (LUM), Complement C7 (C7), Ectonucleotide Pyrophosphatase/Phosphodiesterase 2 (ENPP2), Alpha-2-Glycoprotein 1, Zinc-Binding (AZGP1), Complement C3 (C3), Ceruloplasmin (CP), Transferrin (TF), Complement C4B (C4B), Inter-Alpha-Trypsin Inhibitor Heavy Chain 4 (ITIH4), Apolipoprotein E (APOE), Apolipoprotein A1 (APOA1) and Plasminogen (PLG). The other 21 genes were found to be significant in CSF only (ROC AUC>0.7 in targeted proteomics data but not in transcriptomic data, depicted in circle in **Figure 4A**): Apolipoprotein D (APOD), Serpin Family C Member 1 (SERPINC1), Transthyretin (TTR), Fibrinogen Alpha Chain (FGA), Orosomucoid 2 (ORM2), Immunoglobulin Kappa Constant (IGKC), GC Vitamin D Binding Protein (GC), Phospholipid Transfer Protein (PLTP), Complement C2 (C2), Albumin (ALB), Alpha-2-Macroglobulin (A2M), Fibulin 1 (FBLN1), Orosomucoid 1 (ORM1), Apolipoprotein H (APOH), Immunoglobulin Heavy Constant Gamma 2 (IGHG2), Afamin (AFM), Beta-2-Microglobulin (B2M), Protein S (PROS1), Immunoglobulin Heavy Constant Gamma 4 (IGHG4), Histidine Rich Glycoprotein (HRG) and Complement C8 Beta Chain (C8B).

In addition, we compared the contributions of potential tissue leakage biomarkers (n=18, significant in tissues) and host response biomarkers (n=21, significant in CSF only) to the explained variance, as shown in **Figure 4B**. The explained variance score for the total 39 features was 0.586, while the scores for the features from tissue and host response were 0.580 and 0.556, respectively. Both potential tissue origin features and host response features demonstrated a similar explained variance score, suggesting that these two groups contributed equally to the model. Furthermore, we observed that the explained variance score for all 39 features was comparable between both groups. This suggests that the overall performance of stratification can be achieved by utilizing either the group of proteins derived from tissues or the host response. This was consistent with the observation of the stratification performance of potential tissue leakage biomarkers and host response biomarkers. ROC AUC of these two groups were 0.939 (95%CI: 0.812-0.980) and 0.925 (95%CI: 0.802-0.980) respectively, as shown in **Supplementary Figure 7**.

**Figure 5:**
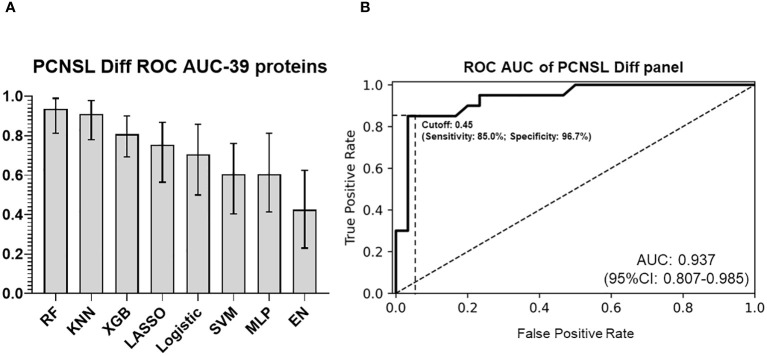
PCNSL differentiation panel with 39 validated proteomic biomarkers **(A)** Comparison of ROC AUC performance for PCNSL differentiation stratification across various modeling algorithms. **(B)** ROC AUC curve for the final 39-protein panel for PCNSL diagnosis, utilizing the random forest algorithm. The abbreviations MLP, RF, KNN, XGB, SVM, and EN represent multilayer perceptron, random forest, k-nearest neighbors, XGBoost, support vector machine, and elastic net linear regression, respectively. Dashed lines showed the optimized sensitivity and specificity at the chosen threshold 0.45.

To develop a CSF-based differentiation model for PCNSL, we used a panel of 39 validated differentially expressed protein biomarkers and compared various algorithms to optimize stratification performance (with “leave-one-out” cross validation). The RF model of the 39 biomarkers panel exhibited the highest ROC AUC (0.937; 95%CI: 0.807-0.985) for distinguishing PCNSL from other brain tumors, as shown in **Figures 5A, B**. The coefficients for each protein in this panel are shown in **Supplementary Figure 8**. This clearly demonstrated that our 39-biomarker panel could accurately differentiate PCNSL from other brain tumors.

### 3.6 Thresholds determination for best performance to discriminate PCNSL from tumor-free control or other brain tumor

To determine the optimal cutoff value for the two panels (identifying PCNSL from non-malignant disease controls or from other brain tumor), we used Youden’s J statistics (22) to select the optimal predicted probability cut-off. It is the maximum vertical distance between ROC curve and diagonal line, where the idea is to maximize the difference between true positive rate and false positive rate. Youden’s J index combines sensitivity and specificity into a single measure (Sensitivity+Specificity-1) and has a value between 0 and 1. Therefore, the optimal threshold (cut-off value) maximizes the sum of the sensitivity and specificity. For our PCNSL diagnosis panel, we selected a threshold of 0.25 (range 0-1), which yielded a sensitivity of 85.0%, specificity of 77.4%, PPV (positive predictive value) of 70.8%, and NPV (negative predictive value) of 88.9%. For our PCNSL differentiation panel, we selected a threshold of 0.45 (range 0-1), which resulted in a sensitivity of 85.0%, specificity of 96.7%, PPV of 94.4%, and NPV of 90.6%

### 3.7 Pathways analysis of the validated biomarkers

**Figure 6:**
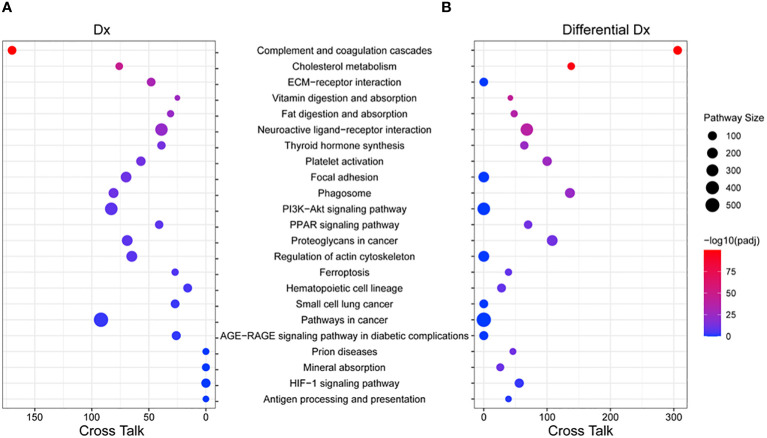
Pathway enrichment and crosstalk analysis of biomarker proteins. **(A)** The significant KEGG pathways that include the 14 proteins from the PCNSL diagnosis panel were shown. **(B)** The significant KEGG pathways that include the 39 proteins from the PCNSL differentiation panel are shown. The size of each circle represents the number of proteins involved in the pathway, and the color represents the pathway’s enrichment p-value. The number of cross talk pathways for each pathway is indicated on the X-axis.

We performed pathway analysis based on the KEGG (v94.1) database using the enrichment and “pathway crosstalk” (PathwAX) method. **Figure 6** shows the top pathways enriched by the 14 protein biomarkers from the diagnosis panel or 39 protein biomarkers from the differentiation panel. Comparing the diagnosis and differentiation panels, some pathways were found to be significant in both panels. The most common significant pathways included, complement and coagulation cascades, cholesterol metabolism, neuroactive ligand-receptor interaction, and platelet activation pathways. Among them, complement and coagulation cascades and cholesterol metabolism also had the greatest number of pathways crosstalk links, which suggested their involvement and interactions with many other cellular activities. Additionally, there were significant pathways that were unique to both the diagnosis panel and differentiation panels. For instance, the diagnosis panel features were significantly enriched in ECM-receptor interaction, focal adhesion, PI3K-Akt signaling, and regulation of actin cytoskeleton pathways. On the other hand, prion disease, mineral absorption and HIF-1 signaling, and antigen-processing and presentation were significantly enriched with differentiation panel features.

## 4 Discussion

In our study, we conducted a reanalysis of the significant proteomic features discovered by Stühler’s group (PMID: 32610669). Their research utilized a shotgun proteomic approach to establish a CSF proteome from patients with various brain tumors, including PCNSL, SCNSL, multiple sclerosis, glioma, and tumor-free controls. To validate these protein biomarkers, we performed a targeted proteomic approach on a validation CSF cohort comprising 30 PCNSL, 30 other brain tumor, and 31 tumor-free control samples. Using a comparative analysis of proteomics and transcriptomics, we developed two models based on validated protein biomarkers. The PCNSL diagnosis panel consisted of 14 protein signatures and achieved an ROC AUC of 0.873 (0.723-0.948) in differentiating PCNSL from tumor-free controls. The PCNSL differentiation panel comprised 39 protein signatures and achieved an ROC AUC of 0.937 (0.807-0.985) in differentiating PCNSL from other brain tumors. Our findings demonstrate the effectiveness of these panels for PCNSL diagnosis and differentiation from other clinical entities.

We did realize that 3 final proteins, GPNMB, VSIG4 and APOC2 from Stühler’s group were not in our two panels. It was due to the high non-detected rate in our enrolled samples, more than 60% of samples in each class showing missing signals. The discrepancy might be due to the difference of LC-MS proteomics profiling setting and difference in mass spectrometry sensitivity between our study and the reference. Another difference between the two studies was the ethics of the study populations. Our study enrolled the East Asia (China) patients, while Stühler’s group enrolled Europe (Germany) patients. To further confirm whether those proteins could be a biomarker for the PCNSL detection in our study, one possibility will be applying other platforms, such as ELISA test of those three proteins on our patients cohort.

In the context of PCNSL diagnosis, it is critical to identify tissue-origin and host response biomarkers for accurate detection, prognosis, and treatment of the disease. Tissue-origin biomarkers are proteins primarily derived from tumor cells, while host response biomarkers are proteins released due to the interaction between tumor cells and the surrounding environment, such as the immune response or other cellular processes. Waldera-Lupa et al. (2017) confirmed that the PCNSL CSF proteome is associated with blood-brain barrier (BBB) dysfunction (23). More than half of the proteins were likely originating from brain tissue, while the remaining proteins were assigned to plasma and liver, indicating that CSF is an ultrafiltrate of blood plasma (24). However, when comparing the CSF data with proteome and transcriptome data from whole-cell lysates of PCNSL tissue, the researchers found that the direct contribution of tumor cells to the PCNSL CSF proteome was quite low. Consistent with those studies, our results similarly observed that only 6 of the 14 proteins, whose expression levels were significantly higher in PCNSL patients than in tumor-free controls, while other proteins were not differentially expressed in tumor tissues between PCNSL and tumor-free controls. This suggests that the majority of potential PCNSL biomarkers, including cytokines (IL-6, IL-10) and soluble receptor proteins, may not stem directly from the tumor tissue. Instead, they could be present in low concentrations within the tumor tissue and become more concentrated within the extracellular space.

Host response biomarkers, on the other hand, could be actively released through classical protein secretion or ectodomain shedding, with most of the peptides from transmembrane proteins originating from the extracellular or luminal domain. In the same study from Waldera-Lupa et al. (2017), the researchers also found changes in the abundance of ectodomains from transmembrane proteins in the CSF of PCNSL patients. This suggested that PCNSL tumor cells actively release metalloproteinases or tissue inhibitors of metalloproteinases, which alter the shedding of tumor cells or cells in the surrounding CNS environment.

Nevertheless, the presence or absence of biomarkers in tumor tissue and CSF depends on several factors, including the specific biological processes involved in the disease, the type and stage of cancer, the sensitivity and specificity of the detection methods used, and the dynamic interplay between different molecular pathways in the tumor microenvironment and the CSF compartment. In general, some biomarkers may be detected in both tumor tissue and CSF because they are secreted by the tumor cells or released from the dying cells into the CSF. These biomarkers may include cytokines, chemokines, and some specific proteins and genes that are associated with lymphoma progression and the immune response. In the case of PCNSL, the presence of these biomarkers in the CSF may reflect the local immune and inflammatory responses to the tumor growth within the central nervous system.

Several biomarkers have been investigated in PCNSL, including cytokine IL-10, which is overexpressed in tumor tissue and detected in the CSF of patients and is associated with the immunosuppressive microenvironment of the tumor, potentially serving as a diagnostic and prognostic biomarker (25). BCL6, a gene involved in B-cell differentiation, is frequently mutated or overexpressed in PCNSL and has been detected in both tumor tissue and CSF (26), but its clinical significance as a biomarker remains unclear. MYD88, involved in the Toll-like receptor signaling pathway, is commonly mutated in PCNSL and has been detected in both tumor tissue and CSF (27, 28), potentially reflecting the CNS-specific immune response to the tumor. While biomarkers from tissue origin or from CSF can offer insights into PCNSL pathogenesis and progression and have clinical implications for diagnosis, prognosis, and treatment monitoring, further studies are needed to validate these biomarkers and investigate their functional roles.

The KEGG database was utilized to perform pathway analysis, revealing both common and unique pathways enriched by protein biomarkers from the diagnosis and differentiation panels. The most significant pathways were those related to complement and coagulation cascades as well as cholesterol metabolism, which exhibited a high degree of pathway crosstalk. While the relationship between complement and coagulation cascades and PCNSL is not fully understood due to limited research in this area, both systems are crucial components of immune responses and inflammation that have the potential to influence cancer development and progression. Abnormal activation of the complement system may promote tumor growth, invasion, angiogenesis, and immune evasion (29), which could be applicable to PCNSL. Similarly, the coagulation system is known to play a critical role in blood clotting and maintaining hemostasis, but is also involved in inflammation, wound healing, and tissue repair. Mounting evidence suggests that the coagulation system may contribute to cancer development and progression by promoting tumor growth, angiogenesis, metastasis, and immune evasion (30). While the direct evidence linking the coagulation system to PCNSL is limited, an imbalance in the coagulation cascade could potentially contribute to the pathogenesis of PCNSL through the promotion of inflammation, angiogenesis, and immune system interactions.

Cholesterol is a critical component of cell membranes and plays a vital role in cellular functions such as cell signaling, membrane fluidity, and steroid hormone synthesis. In PCNSL, disruptions to the BBB have been linked to changes in cholesterol transport, potentially contributing to disease development and progression (31). Furthermore, PCNSL cells have a high demand for cholesterol to produce signaling molecules and synthesize new membranes, and dysregulated cholesterol synthesis may promote cancer cell proliferation (32). Recent studies have highlighted the potential of targeting cholesterol metabolism in treating aggressive and chemo-resistant lymphomas, including PCNSL. Inhibition of cholesterol metabolism-related factors ACAT1 and SR-BI has led to the accumulation of free cholesterol (33), which suppressed lymphoma growth and increased the cytotoxic effects of chemotherapy drugs. The induction of apoptosis in lymphoma cells by SR-BI inhibitors further supports the potential of targeting cholesterol metabolism in PCNSL treatment. Nonetheless, further research is necessary to fully understand the complex interplay between cholesterol metabolism and PCNSL and explore the potential for novel therapeutic strategies targeting this pathway.

A genome-wide expression analysis comparing PCNSL and non-CNS DLBCL revealed that ECM and adhesion-related pathways were among the top altered pathways (34). These findings align with our pathway analysis, which demonstrated significant enrichment of ECM-receptor interaction, focal adhesion, and cytoskeleton regulation pathways in PCNSL patients when compared to tumor-free controls. Another critical differential pathway between these two groups was the PI3K-Akt signaling pathway. This pathway is among the most important human pathways and has been found to be abnormally activated in various tumors, including DLBCL. Recent studies using immunohistochemistry, western blotting, and real-time qPCR have shown significantly higher expression levels of components in the PI3K-Akt-mTOR pathway in PCNSL patients, such as p-AKT, p-mTOR, p-S6 and p-4E-BP1 and mTOR, and this aberrant activation is correlated with poor prognosis (35). Therefore, inhibitors of PI3K-Akt-mTOR signaling have become promising therapeutic targets to improve prognosis (36). While we did observe significant differences in the HIF-1 signaling and antigen processing and presentation pathways between PCNSL and other brain tumors, the underlying roles of these pathways in potential pathological differences require further exploration. HIF-1 plays a major role in the regulation of hypoxia response, and the difference in HIF-1 signaling between PCNSL and other brain tumors suggests a distinct response to the hypoxic microenvironment, which is essential for tumor progression via the activation of angiogenesis, immunosuppression, and metabolic reprogramming (37).

Overall, our study presents a thorough examination of prospective biomarkers for the diagnosis and differentiation of PCNSL from both tumor-free controls and other brain tumors, and identified pathways that provide valuable insights into the molecular mechanisms underlying PCNSL pathology. The verified protein biomarkers and the established models demonstrate great potential for enhancing the accuracy of PCNSL diagnosis in clinical settings.

## 5 Limitations

While this study offers promising insights, it is important to acknowledge some limitations. One limitation is the small sample size used in the proteomic analysis. Additionally, it is important to note that the proteomics and transcriptomics data were generated from different populations and technology platforms. Furthermore, observed transcriptional differences may not necessarily lead to corresponding protein or functional differences. Further investigations are needed to identify these genes and their downstream proteins at the protein level and explore their functions. Despite these limitations, the differences between the proteomics and transcriptomics data can contribute to the overall reliability of the study.

## 6 Conclusions

In summary, this study validated protein biomarkers discovery from a CSF proteomics profiling and developed an accurate clinical test panel for PCNSL diagnosis and differentiation with CSF-based proteomic signatures. It may help overcome the challenges of current diagnostic methods and the precise PCNSL differentiation from other brain tumor would provide indications for a customized treatment strategy to improve patient outcomes.

## Data availability statement

The original contributions presented in the study are included in the article/**Supplementary Material**. Further inquiries can be directed to the corresponding authors.

## Ethics statement

All patients provided written informed consent for the participation in this study. All experiments were conducted in accordance with the Declaration of Helsinki and Good Clinical Practice guidelines and were approved by the Ethics Committee of our institution. The studies were conducted in accordance with the local legislation and institutional requirements. The participants provided their written informed consent to participate in this study.

## Author contributions

JM: Conceptualization, Formal analysis, Methodology, Visualization, Writing – original draft. ZL: Conceptualization, Formal analysis, Visualization, Writing – original draft. YZ: Formal analysis, Investigation, Methodology, Writing – original draft. YD: Conceptualization, Investigation, Methodology, Visualization, Writing – original draft. QT: Conceptualization, Formal analysis, Investigation, Methodology, Visualization, Writing – original draft. YQ: Data curation, Methodology, Writing – original draft. BJ: Data curation, Methodology, Writing – original draft. RL: Writing – review & editing. W-LL: Methodology, Writing – original draft. ST: Methodology, Writing – original draft. ZH: Writing – review & editing, Investigation. CC: Formal analysis, Project administration, Writing – original draft. JS: Writing – original draft. QL: Writing – review & editing. MZ: Writing – review & editing. YL: Writing – review & editing. YM: Writing – review & editing. KS: Writing – review & editing. SN: Writing – review & editing. DM: Writing – review & editing. XL: Conceptualization, Project administration, Resources, Supervision, Writing – original draft, Writing – review & editing. BC: Conceptualization, Funding acquisition, Investigation, Project administration, Resources, Supervision, Writing – original draft.

## Supplementary material

Supplementary Table 1 Protein list of identified biomarker candidates for PCNSL vs. control, and PCNSL vs. other brain tumors.
