# Supplementary material for: Targeted multiplex validation of CSF proteomic biomarkers: implications for differentiation of PCNSL from tumor-free controls and other brain tumors
Source: Front Immunol. 2024 Aug 1;15:1343109. doi: 10.3389/fimmu.2024.1343109 (PMC11322575; doi:10.3389/fimmu.2024.1343109)
Supplement: Supplementary file 1 [file DataSheet_1.docx]

**Supplementary Figure Legends**

**Supplementary Figure 1:**  Boxplots of the candidate biomarker peptides’ concentration in PCNSL and tumor-free controls, using mass spectrometry intensity on the concentration of candidate biomarker peptides.

**Supplementary Figure 2:** Boxplots of the candidate biomarker concentration in PCNSL and tumor-free controls, using normalized gene expression value from microarray of candidate biomarker.

**Supplement Figure 3: ROC AUC curves of potential tissue leakage biomarkers and host response biomarkers for PCNSL diagnosis**. Biomarkers ROC AUC was plotted based on logistic regression model for PCNSL diagnosis using (A) potential tissue leakage proteins (significant in both CSF and tissue) and (B) host response proteins (significant in CSF only).

**Supplementary Figure 4: Coefficients of 14 biomarkers from PCNSL Dx panel:** Logistic regression model coefficient values of 14 biomarkers in the PCNSL diagnosis panel are shown in bar graph. * Proteins that have a corresponding gene in the microarray and show a ROC AUC ≥ 0.7.

**Supplementary Figure 5:**  Boxplots of the candidate biomarker peptides’ concentration in PCNSL and other brain tumor, using mass spectrometry intensity on the concentration of candidate biomarker peptides.

**Supplementary Figure 6:** Boxplots of the candidate biomarker concentration in PCNSL and other brain tumor, using normalized gene expression value from microarray of candidate biomarker.

**Supplement Figure 7: ROC AUC curves of potential tissue leakage biomarkers and host response biomarkers for PCNSL differentiation**. Biomarkers ROC AUC was plotted based on Random Forest model for PCNSL differentiation using (A) potential tissue leakage proteins (significant in both CSF and tissue) and (B) host response proteins (significant in CSF only).

**Supplementary Figure 8:** Random Forest model coefficient values of 39 biomarkers in the PCNSL diagnosis panel are shown in bar graph. * Proteins that have a corresponding gene in the microarray and show a ROC AUC ≥ 0.7.

**Supplementary Figure 1:**  Boxplots of the candidate biomarker peptides’ concentration in PCNSL and tumor-free controls, using mass spectrometry intensity on the concentration of candidate biomarker peptides.

**
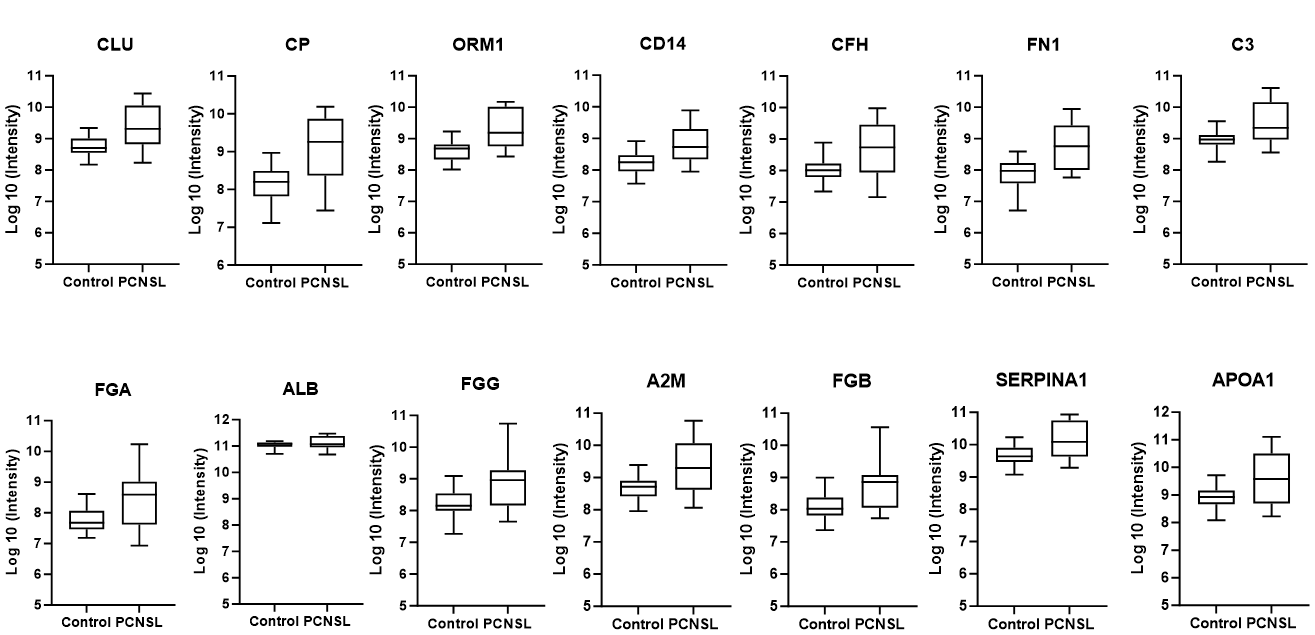
**

**Supplementary Figure 2:** Boxplots of the candidate biomarker concentration in PCNSL and tumor-free controls, using normalized gene expression value from microarray of candidate biomarker.

**
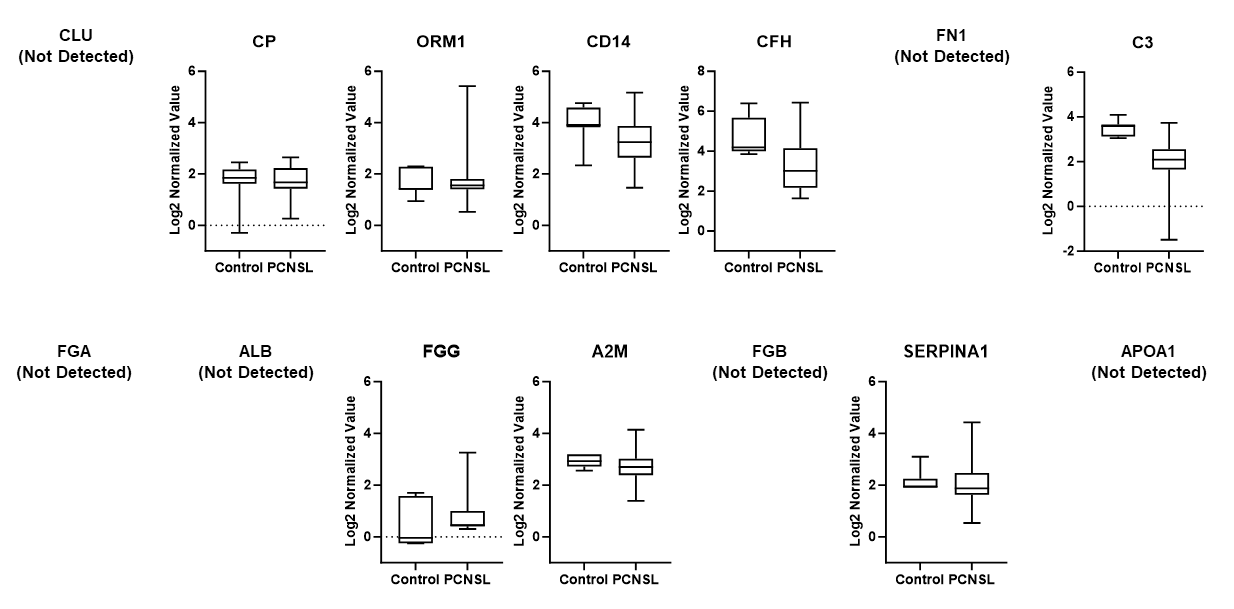
**

**Supplement Figure 3: ROC AUC curves of potential tissue leakage biomarkers and host response biomarkers for PCNSL diagnosis**

**
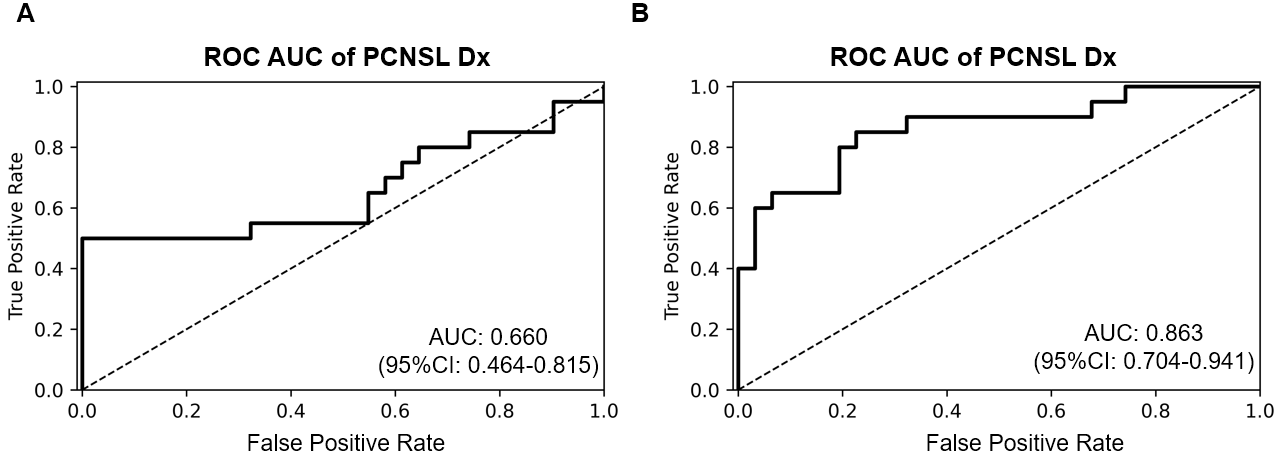
**

**Supplementary Figure 4: Coefficients of 14 biomarkers from PCNSL Dx panel**

**
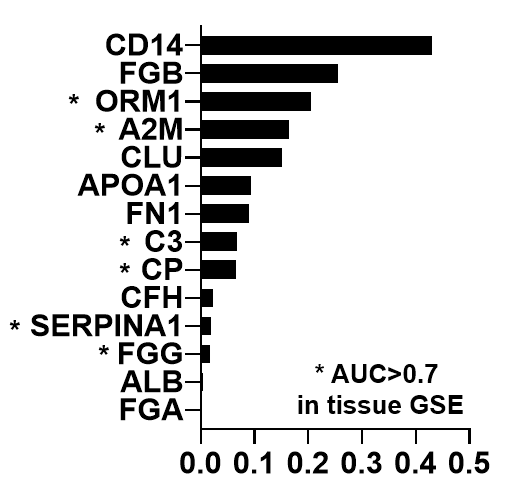
**

**Supplementary Figure 5:**  Boxplots of the candidate biomarker peptides’ concentration in PCNSL and other brain tumor, using mass spectrometry intensity on the concentration of candidate biomarker peptides.

**
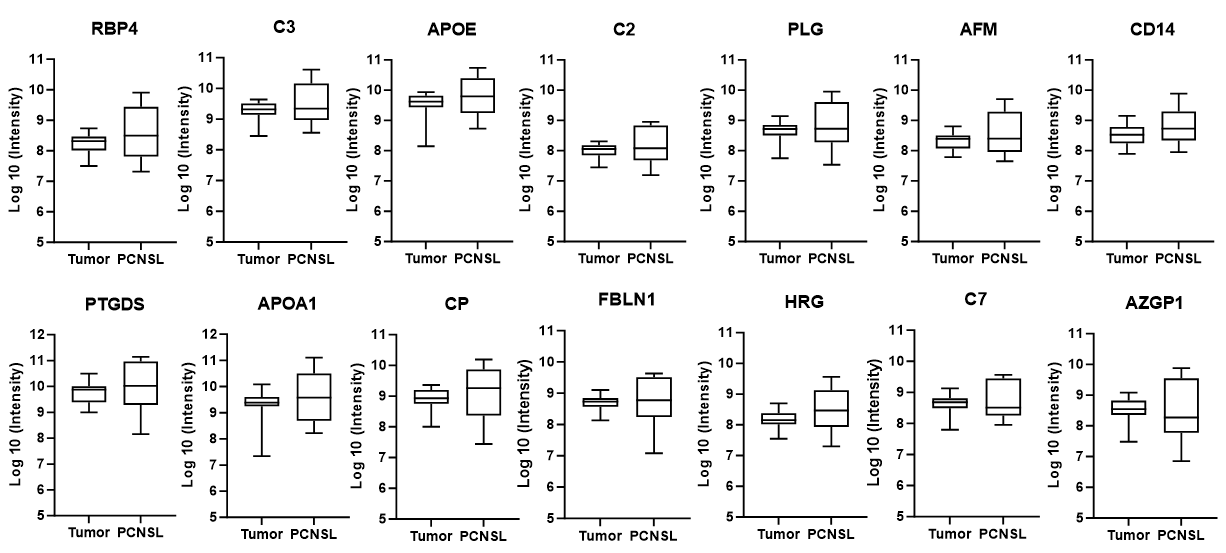
**

**
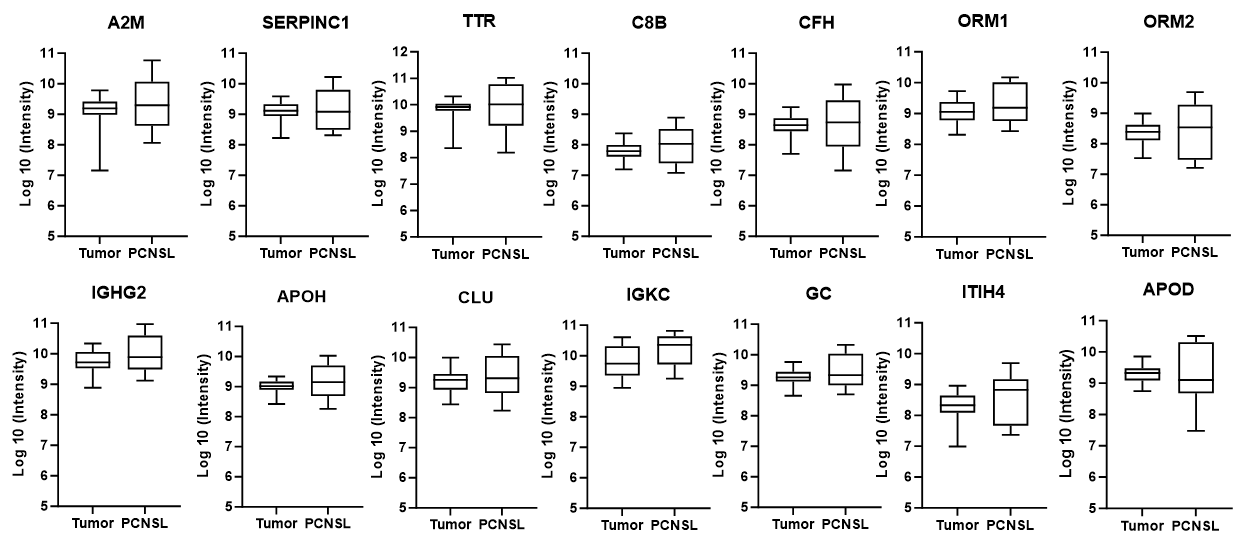
**

**
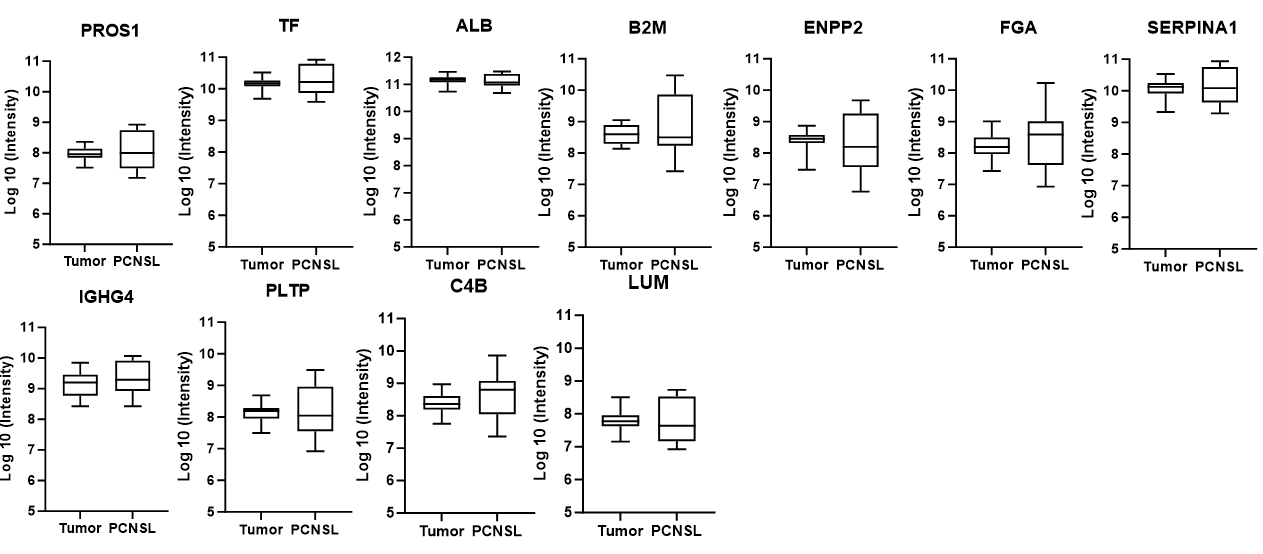
**

**Supplementary Figure 6:** Boxplots of the candidate biomarker concentration in PCNSL and other brain tumor, using normalized gene expression value from microarray of candidate biomarker.

**
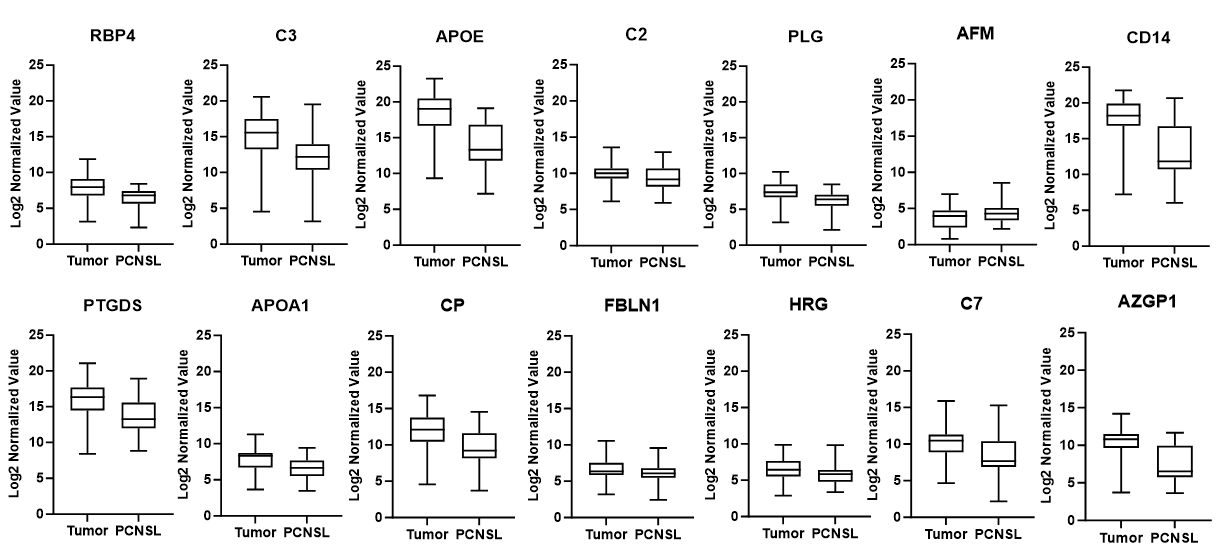
**

**
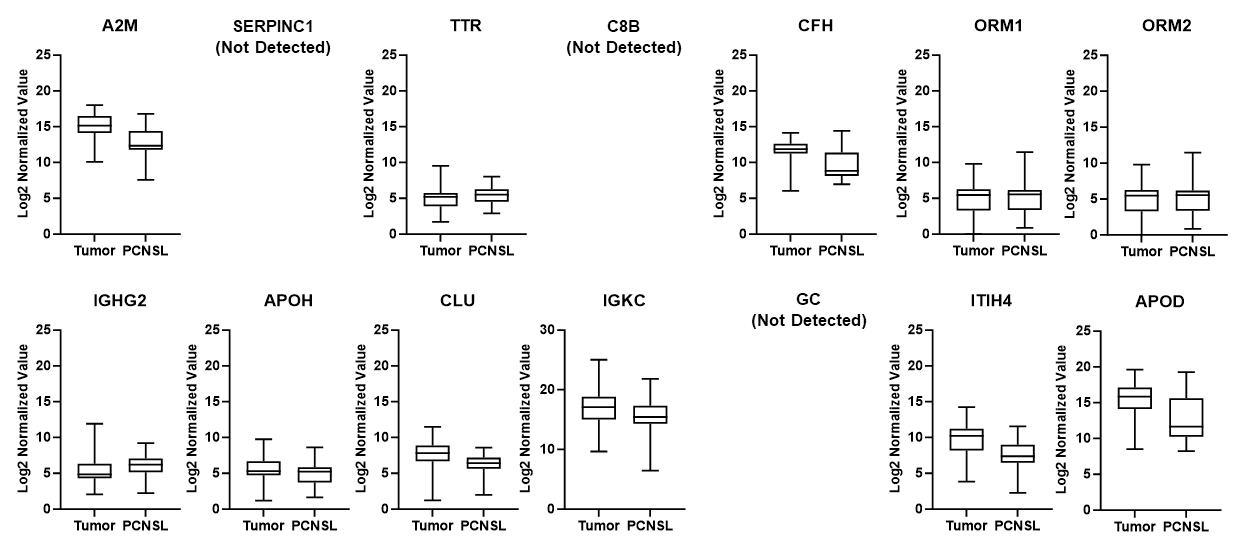
**

**
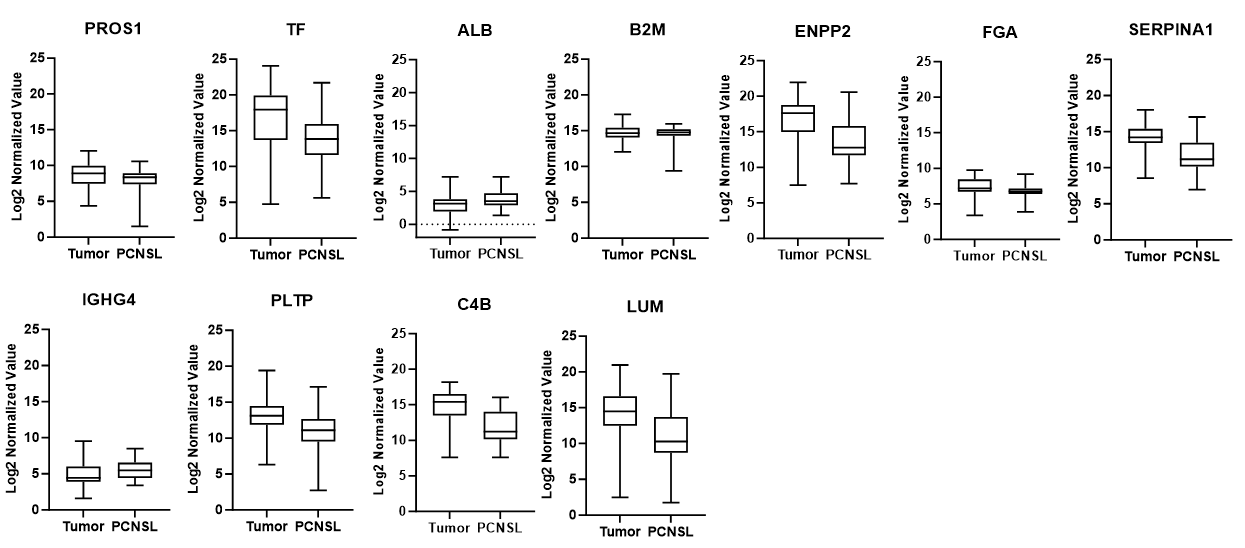
**

**Supplement Figure 7: ROC AUC curves of potential tissue leakage biomarkers and host response biomarkers for PCNSL differentiation**

**
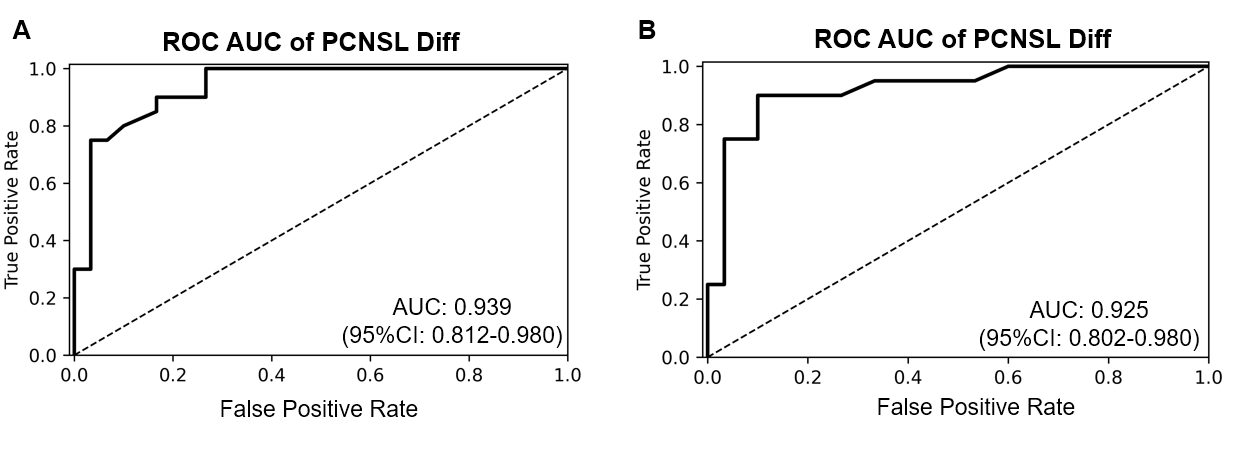
**

**Supplementary Figure 8: Coefficients of 39 biomarkers from PCNSL Dx panel**

**
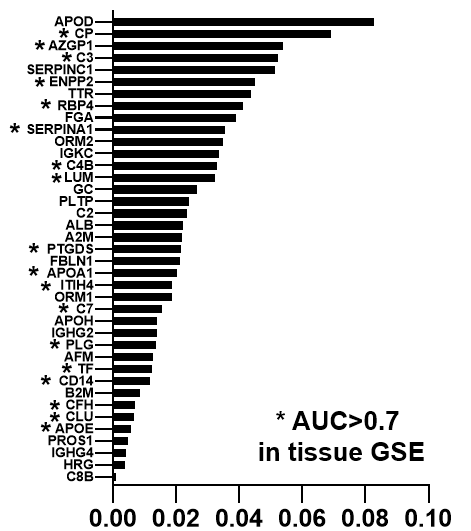
**
